# Social isolation during puberty affects female sexual behavior in mice

**DOI:** 10.3389/fnbeh.2014.00337

**Published:** 2014-09-29

**Authors:** Jasmina Kercmar, Stuart A. Tobet, Gregor Majdic

**Affiliations:** ^1^Veterinary Faculty, Center for Animal Genomics, University of LjubljanaLjubljana, Slovenia; ^2^Department of Biomedical Sciences, Colorado State UniversityFort Collins, CO, USA; ^3^Institute of Physiology, Medical School, University of MariborMaribor, Slovenia

**Keywords:** mice, social isolation, female sexual behavior, estrogen receptor α, puberty/adolescence

## Abstract

Exposure to stress during puberty can lead to long-term behavioral alterations in adult rodents coincident with sex steroid hormone-dependent brain remodeling and reorganization. Social isolation is a stress for social animals like mice, but little is known about the effects of such stress during adolescence on later reproductive behaviors. The present study examined sexual behavior of ovariectomized, estradiol and progesterone primed female mice that were individually housed from 25 days of age until testing at approximately 95 days, or individually housed from day 25 until day 60 (during puberty), followed by housing in social groups. Mice in these isolated groups were compared to females that were group housed throughout the experiment. Receptive sexual behaviors of females and behaviors of stimulus males were recorded. Females housed in social groups displayed greater levels of receptive behaviors in comparison to both socially isolated groups. Namely, social females had higher lordosis quotients (LQs) and more often displayed stronger lordosis postures in comparison to isolated females. No differences between female groups were observed in stimulus male sexual behavior suggesting that female “attractiveness” was not affected by their social isolation. Females housed in social groups had fewer cells containing immunoreactive estrogen receptor (ER) α in the anteroventral periventricular nucleus (AVPV) and in the ventromedial nucleus of the hypothalamus (VMH) than both isolated groups. These results suggest that isolation during adolescence affects female sexual behavior and re-socialization for 1 month in adulthood is insufficient to rescue lordosis behavior from the effects of social isolation during the pubertal period.

## Introduction

Puberty is a period during which an individual attains sexual maturity following the re-activation of the hypothalamic-pituitary-gonadal (HPG) axis and elevated secretion of gonadal steroid hormones (Sisk and Zehr, 2005; Schulz and Sisk, 2006). During puberty, the brain undergoes remodeling and reorganization which is partially influenced by gonadal steroid hormones (Schulz et al., 2009). There is growing evidence that exposure to stressors in adolescence can cause profound long-lasting alterations in the brain and subsequently in behavior in adulthood, perhaps due to interactions between sex hormones and hypothalamic-pituitary-adrenal (HPA) function (McCormick and Mathews, 2007; McCormick et al., 2010; Blaustein and Ismail, 2013). In social mammals like mice and rats, isolation can be stressful (Dixon, 2004; Koolhaas, 2010). Post-weaning social isolation behavioral studies have frequently been done in males, although some studies performed in both sexes suggest differences in the effects of social stress during adolescence, with males appearing to be more vulnerable (reviewed in Blanchard et al., 2001; Fone and Porkess, 2008).

Female sexual behavior is a complex set of behaviors that are necessary and sufficient to achieve fertilization of female ova by male sperm (Nelson, 2005). Two types of female sexual behaviors are often distinguished in rodents: receptive (reflexive postural changes at copulation - lordosis) and proceptive (attracting and initiating copulation) behaviors (Crusio et al., 2013). However, proceptive behaviors are more pronounced in rats than in mice and are difficult to evaluate in mice (Nelson, 2005). Female sexual behavior is mostly regulated by action of sex steroid hormones and neurons in many brain regions sensitive to the action of ovarian steroid hormones (mainly estradiol) are thought to be involved in the regulation of female sexual behavior including those in the preoptic area and ventromedial hypothalamic nuclei (Flanagan-Cato and McEwen, 1995; Rissman et al., 1999; Musatov et al., 2006). The estrogen receptor (ER) α present in neurons in these regions is essential for the effects of estradiol on the expression of sexual receptivity (Rissman et al., 1999). The medial amygdala (MeA) is also rich in estradiol receptors and is another region that may be involved in the regulation of female sexual behavior (DiBenedictis et al., 2012). For example, Fos expression was much higher in the MeA of mated than non-mated females (Flanagan-Cato and McEwen, 1995). ERα can be autoregulated with estradiol down-regulating the expression of ERα in many brain areas (e.g., Simerly and Young, 1991; DonCarlos et al., 1995; Gréco et al., 2001). Stress activates the HPA axis and this can suppress HPG axis activity. Stressed animals may therefore be exposed to lower levels of gonadal hormones (reviewed in Kalantaridou et al., 2004) and this could cause an increase in ERα expression and detection of immunoreactive ERα.

In female mice, some stressors such as LPS injections during the peripubertal period (around 42 days of age) caused reductions in the level of receptive behavior lordosis quotient (LQ). This effect was stronger if mice were stressed peripubertally than if they were stressed in adult life (Laroche et al., 2009a,b). However, not all stressors were equally effective in decreasing receptive behaviors. For example, restraint stress and food deprivation did not have strong effects on these behaviors (Laroche et al., 2009a). The current study reports on the influence of social isolation during pubertal period on sexual behavior in adult female mice, and whether social re-housing in adulthood could eliminate behavioral alterations provoked by social isolation during this vulnerable adolescent period. Sexual behavior was examined in female mice that were group housed, socially isolated (from 25 days of age onwards), or isolated only during the pubertal period (25–60 days of age) followed by group housing. The goal was to explore whether the social isolation during pubertal period might have long-lasting effects on sexual behavior in adult female mice and on the expression of ERα in brain regions important for the regulation of female sexual behavior.

## Materials and methods

### Animals

C57BL/6J mice were originally obtained from Harlan (Italy) and bred at the University of Ljubljana, Veterinary Faculty, in standard conditions with 12-12 light/dark cycle (lights on at 3 am and off at 3 pm) and food (phytoestrogen free diet; Harlan Teklad Diet 2016, Harlan, Milan, Italy) and water *ad libitum*. Mice were weaned at 21 days of age and at 25 days of age females were divided into three groups:

1. group-housed with at least 3 mice per cage (Social),
2. socially isolated with 1 mouse per cage (Isolated),
3. socially isolated and regrouped at 60 days of age (Isol/Social).

Social females (Social, *n* = 8) were housed in larger 15 cm high cages with floor area of 37.5 × 22 cm, socially isolated females (Isolated, *n* = 8) in smaller 14 cm high cages with 35 × 15 cm floor area, and the transiently isolated females (Isol/Social, *n* = 8) first in smaller cages and after regrouping in larger cages. Sexually experienced stimulus males of the same strain (*n* = 9) were individually housed in 13 cm high cages with 28.5 × 10.5 cm floor area that had been previously used for mating (at least 3 successful matings with weaned litters). The C57BL/6J mice were chosen because this is a commonly used reference strain for behavioral phenotyping studies with high rates of copulatory behaviors displayed (reviewed in Crawley et al., 1997).

All animal experiments were approved by the Veterinary Administration of the Republic of Slovenia and were done according to ethical principles, EU directive, and NIH guidelines.

### Surgery and hormonal treatments

All female mice were ovariectomized bilaterally at 60 days of age (after puberty) to eliminate endogenous gonadal steroids. Mice were anesthetized with the mixture of ketamine (Vetoquinol Biowet, Gorzowie, Poland; 100 μg/g BW), acepromazine (Fort Dodge Animal Health, Fort Dodge, IA, USA; 2 μg/g BW) and xylazine (Chanelle Pharmaceuticals Ltd., Loughrea, Ireland; 10 μg/g BW) and gonads were excised through small incisions. The incisions were stitched (absorbable sutures; Safil, Braun, Aesculap, Tuttlingen, Germany) and mice received two injections of butorfanol (Turbogesic, Fort Dodge Animal Health, Fort Dodge, IA, USA; 2 μg/g BW) after surgery to alleviate potential pain. To regulate circulating estradiol levels in adulthood at approximately 80 days of age mice received subcutaneous implants containing estradiol benzoate. Silastic implants (1.02 mm inner diameter, 2.16 mm outer diameter) were filled 5 mm in length with crystalline β-estradiol 3-benzoate (EB; Sigma), diluted 1:1 with cholesterol (Sigma) (Wersinger et al., 1999) and closed on both ends by medical silastic adhesive (Dow Corning). Implants were inserted subcutaneously in the midscapular region under anesthesia (mixture of ketamine (Vetoquinol Biowet, Gorzowie, Poland; 100 μg/g BW), acepromazine (Fort Dodge Animal Health, Fort Dodge, IA, USA; 2 μg/g BW) and xylazine (Chanelle Pharmaceuticals Ltd., Loughrea, Ireland; 10 μg/g BW). These implants yield plasma estradiol levels close to the physiological range normally observed during estrus (Wersinger et al., 1999). Behavior tests were performed at least 10 days after implantation. Approximately 4–8 h before each test the females were injected subcutaneously with 0.8 mg of progesterone (P; Sigma) dissolved in corn oil (Sigma). All mice were initially tested for sexual behavior between 90 and 100 days of age, and were sacrificed by transcardial perfusion fixation with 4% paraformaldehyde 4 days after the last test, around 125 days of age, and the brains were dissected and stored in 0.1 M PB at 4°C until further processing for immunohistochemistry.

### Female sexual behavior test

Female sexual behavior tests were performed in clear glass aquaria (17 cm high with 41.5 × 26 cm floor area) with a mirror positioned under the testing arena to obtain better views of facets of sexual behaviors (Wersinger et al., 1997). Females were tested during the first 2–4 h of the dark period of the circadian cycle, under dim red light illumination, and the test sessions were videotaped for subsequent scoring. Each female was tested 6 times, every 4–5 days to mimic the normal physiological estrus cycle. The first trial served for animals to gain sexual experience prior to testing, the next five trials were scored. The stimulus males were placed into the aquarium at least 4 h prior behavior testing with at least 3 day old bedding and food and water *ad libitum* to acclimate to the novel environment. Food and water were removed during the behavior tests.

Hormonally-primed females were placed in the middle of aquaria with a stimulus male for 20 min (Park, 2011), or until the female received an ejaculation. The following behaviors were recorded: lordosis posture, total number and latency of attempted mounts, successful mounts, pelvic thrusts and intromissions, and the latency of ejaculation. Behaviors were recorded by “stopwatch” software (Center for Behavioral Neuroscience, Atlanta, GA) and were observed by the same investigator (Jasmina Kercmar) who was blinded to the group assignment at the time of testing.

If the stimulus male did not try to mount the female, after 5 min of testing the tested female was moved to another aquaria with a new, previously habituated stimulus male. Ejaculating males were not used again for the remainder of the trial day. Mounts were counted when the female had all four limbs on the floor. Female lordosis posture as an index of sexual receptivity was scored from 0 (no receptive behavior with no lordosis reflex) to 5 (completely receptiveness with strongest lordosis reflex as described previously (Bakker et al., 2002). Lordosis was defined using the following stipulations: all four paws are grounded, hind region is elevated off the floor of the test chamber, and the back is slightly arched (Takasugi et al., 1983; Kudwa et al., 2007). A LQ was calculated by the following formula: number of mounts during which the female stood still (lordosis 4 and 5)/total number of attempted and successful mounts × 100.

### ERα immunohistochemistry

Brains were embedded in 5% agarose (Sigma) and sectioned at 50 μm in cold 0.05 M PBS using a vibrating microtome (Integraslice 7550 MM, Campden Instruments, UK). Sections were incubated in 0.1 M glycine (Sigma) in cold 0.05 M PBS for 30 min followed by incubation in 0.5% sodium borohydride (Sigma) for 15 min at 4°C. Glycine and sodium borohydride were washed out with 15 min and 20 min washes (every 5 min) in cold 0.05 M PBS. Sections were blocked in 5% normal goat serum (Jackson Immunoresearch, West Grove, PA, USA) containing 0.5% Triton X-100 (Sigma) and 1% H_2_O_2_ (Merck, Darmstadt, Germany) for 30 min at 4°C. Rabbit primary antiserum against ERα (1:5000, Cat.#06-935, Upstate, Lake Placid, NY, USA) were diluted in 0.05 M PBS containing 1% bovine serum albumin (Sigma) and 0.5% Triton X-100. Sections were incubated with primary antibodies over 2–3 nights at 4°C with shaking. Sections were after then washed in 0.05 M PBS containing 1% normal goat serum and 0.02% Triton X-100 four times for 15 min at room temperature. Biotinylated secondary antibodies (Jackson Immunoresearch) against primary rabbit antiserum were diluted 1:500 in 0.05 M PBS containing 1% normal goat serum and 0.5% Triton X-100. Sections were incubated with biotinylated secondary antibodies for 2 h, followed by 4 washes for 15 min in 0.05 M PBS buffer containing 0.02% Triton X-100. Streptavidin–HRP complex (Jackson ImmunoResearch) was diluted 1:2500 in 0.05 M PBS solution containing 0.5% Triton X-100. Sections were incubated with Streptavidin–HRP for 1 h at room temperature and then washed in Tris-buffered saline (0.05 M Tris–HCl/0.9% NaCl; pH 7.5; Sigma) for 1 h (four times for 15 min) at room temperature. Antigen–antibody complexes were visualized as a black reaction product by incubating sections in 0.025% 3,3′-diaminobenzidine/0.2% ammonium nickel (II) sulfate substrate (Sigma) in Tris-buffered saline containing 0.02% H_2_O_2_ for 5 min at room temperature. Sections were finally washed in Tris-buffered saline three times every 10 min. After mounting, sections were dried and coverslipped using hydrophobic medium (Pertex; Burgdorf, Germany).

### Data collection and ERα quantification

Digital images of brain regions of interest were obtained using a Nikon Eclipse 80i microscope with Nikon DS-Fi1 camera. Images were enhanced for contrast using Adobe Photoshop software (Version 8.0). The number of immunoreactive cells or total area that was immunoreactive for ERα was analyzed in coronal sections containing the anteroventral periventricular region (AVPV) between 0.26 and 0.14 mm rostral from Bregma, the ventromedial hypothalamic region (VMH) 1.70 mm caudal from Bregma, and MeA 1.22 mm caudal from Bregma according to stereotaxic coordinates (Franklin and Paxinos, 2008). All digital images were taken under 100x magnification. The base of the brain was considered as a reference boundary with the third ventricle in the center of the images for AVPV, the third ventricle and the base of the brain as reference boundaries for VMH, and the junction of the optic nerve and cortex-amygdala transition as reference boundary with this junction in the middle of the lateral sides of the images for MeA. Due to the possibility of asymmetry in antigen detection in VMH and MeA between the left and right sides of the brain, the side (unilateral) with more immunopositive cells was always chosen for analysis. Immunoreactivity in AVPV was assessed on both sides (bilateral) of the third ventricle, extending approximately 640 μm laterally from the third ventricle and 960 μm dorsally from the base of the brain. Due to the large number of overlapping immunopositive ERα cells in AVPV, the immunoreactive area was quantified using custom software (Surfkvad; made by Dr. Marko Kreft, Institute of pathophysiology, Faculty of Medicine, Ljubljana) that divides an image into 6 × 8 squares (measuring 160 × 160 μm each under 100x magnification) and calculates a percentage of dark area for each square (Büdefeld et al., 2008). To standardize the collection of immunoreactive area data, all images were taken under the same illumination and converted to grayscale. Grayscale images were subjected to threshold conversion to selectively identify immunoreactive elements using Photoshop software. Black and white images were then analyzed with Surfkvad. The number of immunopositive cells in the VMH and MeA was counted with the help of Image J software (NIH, Bethesda, MD). The grayscale images were divided into a grid of 6 × 8 squares for VMH (measuring 160 × 160 μm each), and a grid of 8 × 10 squares for MeA (measuring 120 × 120 μm each ). Only the grid delimiting the VMH region (4 × 4 squares; extending approximately from 480 μm to 1120 μm laterally from the third ventricle and 640 μm dorsally from the base of the brain), and the grid delimiting the MeA region (3 × 3 squares; extending approximately from 120 μm to 480 μm laterally, 120 μm ventrally and 240 μm dorsally from the lateral boundary) were analyzed (Figures 2B,C).

**Figure 1:**
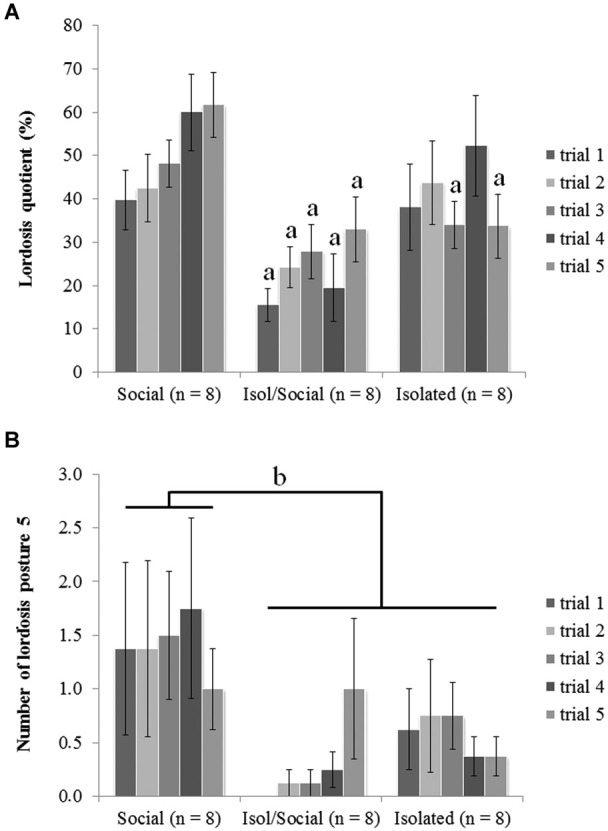
**Mice that were socially isolated during puberty showed impaired receptive female sexual behavior**. **(A)** LQ (^a^ *p* < 0.01), **(B)** number of displayed lordosis posture scored with 5 (^b^ *p* < 0.05). Data are reported as mean ± SEM; ^a, b^ Significant difference between females housed in social groups (Social) and other two isolated groups (Isol/Social, Isolated).

**Figure 2:**
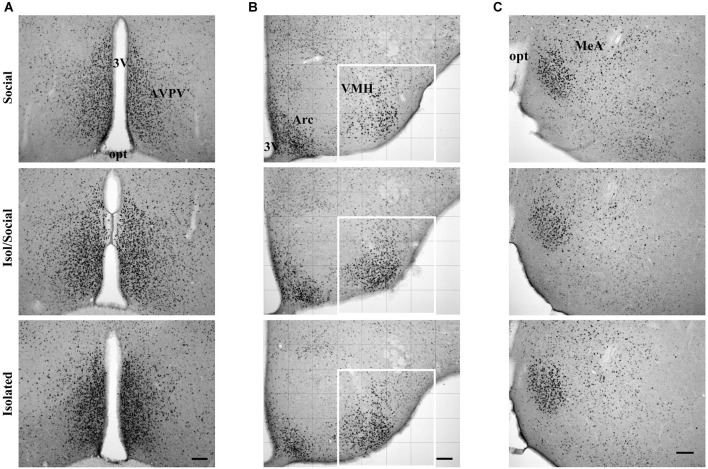
**Digital images showing ERα immunoreactive cells**. **(A)** In the anteroventral paraventricular nucleus (AVPV), **(B)** ventromedial nucleus of the hypothalamus (VMH) and **(C)** in the medial amygdala (MeA) in socially housed mice (Social), isolated for transient time (Isol/Social) and in isolated throughout the experiment (Isolated). In the VMH images, the white square denotes the area analyzed. Bar = 100 μm. 3V—third ventricle, opt—optic nerve, Arc—arcuate nucleus.

### Statistical analyses

All data were statistical analyzed using NCSS software (NCSS statistical software, Kaysville, UT, USA). To test differences between groups in sexual behavior tests, repeated measures ANOVA was performed with housing condition as independent variable, and trial as a repeated measure (within) factor, followed by *post hoc* Fisher LSD tests. Eight mice in each group were tested for female sexual behaviors (Social, *n* = 8; Isolated, *n* = 8; Isol/Social, *n* = 8). Differences between groups in the number or area of immunoreactive ERα were analyzed by ANOVA followed by Fisher LSD *post hoc* (for VMH and amygdala) and by repeated measures ANOVA followed by Fischer LSD *post hoc* test (for AVPV). At least 4 brains in each group were analyzed and differences were considered statistically significant at *p* < 0.05.

## Results

### Sexual behavior of female mice

Social isolation during adolescence reduced female sexual behaviors, and re-socialization in adulthood was insufficient to rescue receptive lordosis behavior from the effects of social isolation during the pubertal period. ANOVA revealed a significant overall effect of housing condition [*F*_(3, 24)_ = 7.57, *p* < 0.01] on the LQ between all three groups (Figure 1A). The *post hoc* tests indicated that mice housed in social groups (Social) had higher LQs in comparison with mice isolated for the limited period (Isol/Social) in all five trials while mice isolated throughout the experiment differ from the socially housed group selectively in trials 3 and 5.

ANOVA also showed significant effect of housing condition on the number of displayed lordosis reflexes scored 5 (the highest receptiveness with strongest lordosis reflex) [*F*_(3, 24)_ = 5.32, *p* < 0.05] (Figure 1B). *Post hoc* analysis showed that socially housed mice (Social) displayed lordosis reflexes scores of 5 more often than did other two groups of female mice (Isol/Social, Isolated). There were no statistically significant differences between permanently isolated mice and mice isolated only during the pubertal period, suggesting that 1 month re-socialization could not rescue from the effects of social isolation during puberty.

### Sexual behavior of stimulus male mice

No differences between female groups (Social, Isol/Social and Isolated) were observed in stimulus male sexual behavior, suggesting that female “attractiveness” was not affected by social isolation.

Repeated measures ANOVA did not show any significant effect of housing conditions (Social, Isol/Social and Isolated; means ± SEMs of all five trials) in the total number of mounts (19.1 ± 2.2, 19.9 ± 1.9 and 13.7 ± 1.4), thrusts (326.3 ± 33.1, 299.0 ± 30.3 and 268.3 ± 26.3), or intromissions (16.7 ± 2.0, 15.5 ± 1.7 and 11.8 ± 1.3) nor in the latencies to mount (54.8 ± 9.1, 56.4 ± 7.7 and 69.9 ± 12.0), intromit (82.8 ± 14.4, 112.6 ± 17.4 and 109.7 ± 17.2), or ejaculate (793.9 ± 65.9, 923.1 ± 60.2 and 767.5 ± 68.9).

### Expression of ERα in AVPV, VMH and MeA

**Figure 3:**
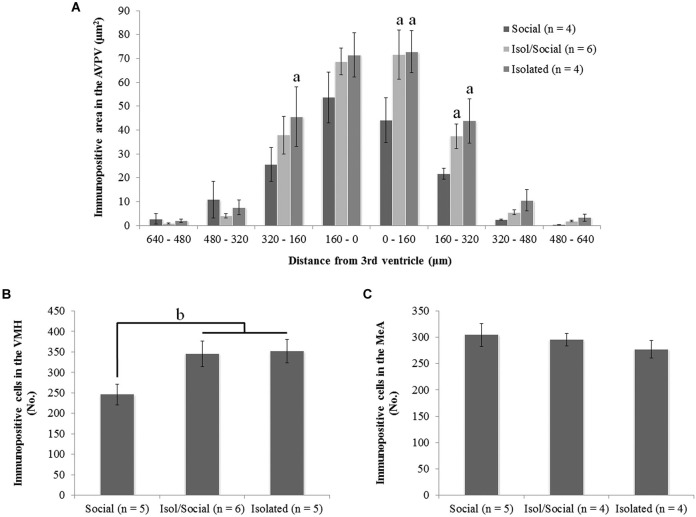
**Social isolation during puberty increases ERα immunoreactivity in the anteroventral paraventricular nucleus (AVPV) and in the ventromedial nucleus of the hypothalamus (VMH), but not in the medial amygdala (MeA). (A)** Area of representing immunopositive ERα cells in the AVPV on both sides of the third ventricle (^a^ *p* < 0.01), **(B)** total number of immunopositive ERα cells in the VMH (^b^ *p* < 0.05), **(C)** total number of immunopositive ERα cells in the MeA. Data are reported as mean ± SEM; ^a^ Significantly different from females housed in social groups (Social). ^b^ Significant difference between females housed in social groups (Social) and other two isolated groups (Isol/Social, Isolated).

Statistically significant differences in ERα immunoreactivity were found in the AVPV, VMH, but not in the MeA (Figures 2, 3). Socially housed mice had less ERα immunoreactive area than isolated mice of both groups. Repeated measures ANOVA with columns as the within factor showed a significant effect of housing condition in the total immunoreactivity for ERα in cells in the AVPV [*F*_(3, 14)_ = 6.72, *p* < 0.01] (Figures 2A, 3A). ANOVA revealed a significant effect of housing condition in the number of ERα immunoreactive cells in the VMH [*F*_(3, 16)_ = 3.93, *p* < 0.05] (Figures 2B, 3B), but not in the MeA (Figures 2C, 3C). *Post hoc* analysis revealed that socially housed mice (Social) had less ERα immunoreactive area in the AVPV and less ERα immunoreactive cells in the VMH than the other two housing groups (Isol/Social, Isolated; Figures 3A,B). Mice isolated throughout the experiment or only during puberty had more immunoreactive area or more cells in the AVPV and VMH, respectively, than socially housed mice, and there was no statistical difference in total immunoreactivity or number of cells in both AVPV and VMH between permanently isolated mice and mice isolated for transient time only (Figures 3A,B).

## Discussion

Beside the pre- and early postnatal period, the pubertal period is important for the appropriate development of specific behaviors displayed in adulthood (Sisk and Zehr, 2005). Early life stress can have profound influences on brain development and subsequently on behavior later in life (reviewed in McCormick et al., 2010). Particular stressors during the pubertal period in female mice (Laroche et al., 2009a,b; Ismail et al., 2011) may cause enduring changes in behavioral responsiveness of the brain to estradiol and progesterone. The current study provides new information about the effects of a different source of stress, post-weaning social isolation, on female sexual behavior. Sexual behavior was examined in female mice that were individually or group housed from 25 days of age throughout the experiment, or individually housed from day 25 until day 60 (during puberty), followed by housing in social groups. The results of the current study suggest the importance of the social environment during puberty for the display of sexual behaviors in adult female mice.

In the current study socially housed female mice displayed stronger lordosis behavior in comparison to mice that were socially isolated during puberty (Isol/Social or Isolated). There were no significant differences between mice that were isolated during puberty vs. re-socialized afterward. Therefore, 1 month re-socialization was insufficient to rescue the behavior from the deleterious effects of social isolation during puberty. This is consistent with previous reports about sexual behavior in female mice that were exposed to different stressors during the peripubertal adolescent period (Laroche et al., 2009a,b; Ismail et al., 2011). In the current study there was a significant overall effect of housing on LQ. However, *post hoc* tests revealed significant differences between social and social/isol groups in all five trials while the difference in LQ was significantly different between social and isol groups more selectively in trials 3 and 5. This is intriguing, as it suggests a potentially stronger effect of temporary isolation than permanent isolation. Perhaps this could be explained by the possibility that re-socialization after isolation during puberty might present additional stress for female mice, while persistent isolation possibly force mice to adapt to the isolation and in longer period of isolation somehow compensate some of the effects of isolation stress. The observation that isolation stress selectively during puberty had a stronger effect on LQ than isolation throughout the experiment is partially in agreement with the previous study by Laroche and coworkers that reported mice exposed to stress (shipping or LPS injection) at 6 weeks of age (peripubertally) show lower levels of sexual receptivity than mice exposed to the same stressors at 12 weeks of age (in adulthood) or control mice (Laroche et al., 2009a,b). Thus, the pubertal period might be an especially vulnerable period for stress to cause alterations in the circuitry regulating female sex behavior. In contrast, sexual behavior of socially housed or isolated female rats after weaning did not differ (Duffy and Hendricks, 1973), suggesting that female mice might be more vulnerable to social isolation stress during puberty than female rats. As expected, there were too few proceptive behaviors (sniffing or following the male) seen in any female mice to analyze impact. Interestingly, the sexual behaviors of stimulus males (mounts, thrusts, intromissions and ejaculation) were not altered by the different housing regimes of the test females, suggesting that female “attractiveness” was not affected by social isolation during puberty.

Estradiol effects in the brain are mediated via interactions with ERs and for the regulation of female sexual behavior by estradiol, ERα is essential (Ogawa et al., 1998; Rissman et al., 1999). MPOA, MeA, and VMH and other sites are rich in ERs that likely contribute to sexual behaviors (Flanagan-Cato and McEwen, 1995; Rissman et al., 1999; DiBenedictis et al., 2012). In the current study, social isolation (permanent or for a specifically limited period) increased the area of ERα immunoreactivity in the AVPV and the number of ERα immunopositive cells in the VMH in comparison to socially housed mice. This contrasts with previous studies in mice (Ismail et al., 2011) and prairie voles (Ruscio et al., 2009), where females stressed during puberty had reduced numbers of ERα immunopositive cells in different brain areas (MPOA, BNST, VMH and arcuate nucleus) involved in the regulation of female sexual behavior in comparison to control animals. One report (Ismail et al., 2011) showed reduced levels of ERα in the MPOA, VMH and arcuate nucleus of adult mice (at 16 weeks of age), but not in the AVPV, after exposure to shipping stress during the pubertal period at 6 weeks of age. Another report (Ruscio et al., 2009) showed no differences in immunoreactive ERα in the MeA and VMH, but reduced expression in MPOA and BNST, between isolated and pair housed (different sex pairs) prairie voles. These differences among studies may be due to the use of different stressors, the timing of stress, and species or strain differences. All of these factors have been shown to cause differences in stress effects (reviewed in McCormick et al., 2010). Estradiol has been shown to auto-regulate its receptor expression with increased levels of estradiol having suppressive effects on ERα mRNA or protein (DonCarlos et al., 1995; Gréco et al., 2001). In the present study, all mice were exposed to estradiol prior to sacrifice and therefore the differences between groups are presumably due to differences in housing/exposure to social stress. The difference between our study and study by Ismail et al. (2011) which reported reduction of ERα cell numbers in the VMH and no differences in AVPV could be explained by the duration of stress. Namely, in the current study, the stress was prolonged whereas in the prior study in mice (Ismail et al., 2011) the duration of stress was limited and short. Perhaps reduced expression of ERα in certain brain areas presents a rebound effect following short stress and consequent suppression of HPG axis, followed perhaps by a compensatory period with increased estradiol levels. By contrast, prolonged stress could more permanently affect/reduce estradiol levels, causing an increase in receptor expression. Future studies should consider determining the time course of changes in ERα in the relevant brain areas and possible mediating factors.

As noted in the Introduction section, MeA is also thought to be involved in female sexual behavior, since mating increases c-Fos expression in this brain region (Flanagan-Cato and McEwen, 1995; Sah et al., 2003; DiBenedictis et al., 2012). However, another study in female rats suggested that impairment of sexual behavior is not due to ERα knockdown in MeA (Spiteri et al., 2010a), indicating that female sexual behavior is not modulated by the ERα in MeA in rats. As for many characteristics, it is difficult to know whether this is true for rats and mice (Bonthuis et al., 2010). Indeed, in the present study there was not a significant alteration in the number of ERα immunopositive cells in the MeA regardless of different housing regimes during puberty.

The amygdala is a major brain region mediating emotional and hormonal responses to stress (mainly the basolateral complex of amygdaloid nuclei and central nucleus of the centromedial complex) (reviewed in Sah et al., 2003). Regardless of the lack of influence of social isolation on immunoreactive ERα, alterations of lordosis behavior in socially isolated mice in the current study could be due to increased general anxiety or impaired social behaviors. Anxiety-like behaviors were not directly assessed. In the absence of proceptive behaviors or group differences in attractiveness to stimulus males, impaired lordosis behavioris perhaps less likely due to impaired social interactions. In agreement, a study in socially isolated female rats during puberty showed no differences in social interactions in comparison to socially grouped rats (Lukkes et al., 2012). Another study in mice found no differences in aggressive behavior in female mice of different housing conditions (Ouchi et al., 2013). Other studies of housing isolation stress after weaning in female C57BL/6J mice reported no effect on anxiety-like behavior in comparison to socially housed mice (~4 weeks of isolation (Pietropaolo et al., 2008) or ~8 weeks of isolation (Kulesskaya et al., 2011)). Interestingly, one study in socially housed female rats suggested that anxiety-like behavior might be modulated by ERα in the MeA based on reductions of anxiety-like behaviors after silencing ERα in the MeA (Spiteri et al., 2010b). The lack of differences in MeA ERα immunoreactivity in the current study might be taken to suggest that all three groups of females in the current study were in similar anxiety-like states.

In conclusion, the results of the current study suggest that social isolation stress during puberty/adolescence can have a profound effect on female sexual behavior and on the detection of immunoreactive ERα in brain regions important for behavior regulation in adult female mice without impacting female attractiveness to stimulus males. Re-socialization for 1 month in adulthood could not reverse the effects of social isolation during the pubertal period. These results highlight the importance of social environment during puberty on the development of sexual receptivity in female mice and this should be taken into account when planning or interpreting results of such behavior assessment studies.

## Conflict of interest statement

The authors declare that the research was conducted in the absence of any commercial or financial relationships that could be construed as a potential conflict of interest.
